# Extended hepatic metastasectomy for renal cell carcinoma—new aspects in times of targeted therapy: a single-center experience over three decades

**DOI:** 10.1007/s00423-019-01852-4

**Published:** 2020-01-14

**Authors:** Oliver Beetz, Rabea Söffker, Sebastian Cammann, Felix Oldhafer, Florian W. R. Vondran, Florian Imkamp, Jürgen Klempnauer, Moritz Kleine

**Affiliations:** 1 Department of General, Visceral and Transplant Surgery, Hannover Medical School, Carl-Neuberg-Strasse 1, 30625 Hannover, Germany; 2 Clinic for Urology and Urologic Oncology, Hannover Medical School, Carl-Neuberg-Strasse 1, 30625 Hannover, Germany

**Keywords:** Renal cell carcinoma, Non-colorectal liver metastases, Hepatic metastasectomy, Extended surgery

## Abstract

**Purpose:**

Despite the introduction of novel targeted therapies on patients with renal cell carcinoma, syn- and metachronous metastases (including hepatic lesions) are observed frequently and significantly influence patient survival. With introduction of targeted therapies as an effective alternative to surgery, therapeutical strategies in stage IV disease must be reevaluated.

**Methods:**

This is a retrospective analysis of 40 patients undergoing hepatic resection of histologically confirmed RCC metastases at our institution between April 1993 and April 2017.

**Results:**

The interval between nephrectomy for renal cell carcinoma and hepatic metastasectomy was 44.0 months (3.3–278.5). Liver resections of different extents were performed, including multivisceral resections. The median follow-up was 37.8 months (0.5–286.5). Tumor recurrence after resection of hepatic metastases occurred in 19 patients resulting in a median disease-free survival of 16.2 months (0.7–265.1) and a median overall survival of 37.8 months (0.5–286.5). Multivariable analysis identified multivisceral resection as an independent risk factor for disease-free and overall survival (*p* = 0.043 and *p* = 0.001, respectively). A longer interval between nephrectomy and hepatic metastasectomy was identified as an independent significant protective factor for overall survival (*p* < 0.001). Patients undergoing metastasectomy after introduction of sunitinib in Europe in 2006 (*n* = 15) showed a significantly longer overall survival (45.2 (9.1–111.0) versus 27.5 (0.5–286.52) months in the preceding era; *p* = 0.038).

**Conclusion:**

Hepatic metastasectomy, including major and extended resections, on patients with metastasized renal cell carcinoma can be performed safely and may facilitate long-term survival. Due to significant morbidity and increased mortality, multivisceral resections must be weighed against other options, such as targeted therapy.

**Electronic supplementary material:**

The online version of this article (10.1007/s00423-019-01852-4) contains supplementary material, which is available to authorized users.

## Introduction

Renal cell carcinoma (RCC) is a malignant disease with increasing incidence, ranging from 3 to 12 cases per 100,000 people in Europe and Northern America.

Despite entering the era of novel targeted therapies such as tyrosine kinase inhibitors, mortality rates are still at more than 2.4 per 100,000 people in many Western countries [1]. This is partially owed to the fact that around 30% of the patients initially present themselves with synchronous distant metastases, whereas another 30% suffer from metachronous metastases after nephrectomy in curative intent [2]. Although bone and lung lesions are most frequently observed in cases of metastatic disease, the liver is involved in 20% of the patients [3]. Studies in the past have analyzed the influence of metastasectomy in general on the disease-free and disease-specific survival and showed beneficial effects for surgical resection in selected patients [4, 5].

The role of hepatic resection is still a matter of debate: Grimes, Pikoulis, and Pinotti each published reviews on this issue with patient series ranging from 4 to 85 patients [6–8]. However, patients with RCC liver metastases are often included in reports on resection of non-colorectal and non-endocrine liver metastases, limiting the significance of drawn conclusions. Furthermore, none of the studies present significant patient numbers after introduction of targeted therapy.

Several scores have been provided in the past to estimate the prognosis of patients in a metastatic stadium (stadium IV) of RCC. The Memorial Sloan-Kettering Prognostic Factors Model, which was introduced by Motzer et al. in 1999 and modified by Mekhail et al. in 2005, is currently the most widely accepted and validated model for larger patient series [9–11].

The objective of the present study was to validate the latter prognostic model for patients undergoing resection of hepatic metastases and to further evaluate the influence of major and extended liver or multivisceral resection (among other selected variables) on the outcome in this special patient collective.

## Methods

### Study cohort

This is a retrospective analysis of patients with stage IV RCC undergoing surgical resection at the Department of General, Visceral and Transplant Surgery, Hannover Medical School, Germany between April 1993 and April 2017.

### Inclusion criteria

Included were all hepatic resections for histologically confirmed metastases of RCC in patients older than 18 years of age. Patients with concomitant extrahepatic disease were included in cases of resectability. No further exclusion criteria were defined. Two patients were lost to follow-up immediately after discharge and were therefore excluded from further survival analysis.

### Definition of variables

Major hepatic surgery was defined as resection of 3 or more liver segments, whereas extended hepatic surgery was defined as resection of 5 or more segments, based on the Brisbane classification [12]. Multivisceral resection was defined as additional resection of infiltrated extrahepatic tissue.

The patients were stratified according to the modified Memorial Sloan-Kettering Score as introduced by Mekhail et al. in 2005 [10]. Poor prognostic factors summarized to a total score for each patient were time from RCC diagnosis until hepatic metastasectomy less than 12 months, hemoglobin levels below lower limit of reference range, LDH more than 1.5 times above the upper limit of reference range, corrected serum calcium above 10 mg/dl, previous radiotherapy, and more than one metastatic site. Patients were stratified into three groups: favorable (0–1 risk factors), intermediate (2 risk factors), poor (3 or more risk factors) risk groups [10].

Postoperative complications were classified according to Dindo et al., ranging from grade 0 (no complications), grade I (minor deviations), grade II (significant alterations), grade III (requiring interventions), grade IV (life-threatening), to grade V (death) [13].

### Study endpoints

Primary study endpoints were disease-free (DFS) and overall survival (OS) upon resection of RCC liver metastases. DFS was defined as time between hepatic metastasectomy and proof of disease recurrence in the course of follow-up, irrespective of the localization (not limited to hepatic recurrence). Secondary endpoints were surgical complications as defined by Dindo et al. and length of hospital and intensive care unit (ICU) stay.

## Statistical methods

The influence of nominal and ordinal variables on binary study endpoints was analyzed with chi-squared test and Fisher’s exact test. Median and mean values between groups were compared with the Student’s *t* test or Mann-Whitney *U* test. Kaplan-Meier analyses including log-rank tests were performed where appropriate. Risk factors for patient survival were initially analyzed with univariable Cox regression analysis. Identification of independent risk factors influencing DFS and OS was achieved by using purposeful selection of variables with a rate of missing values < 10% and *p* values in univariable Cox regression of < 0.300 and consecutive stepwise forward selection.

The collected data was implemented and analyzed using SPSS statistical software (version 26; SPSS Inc.; IBM Corporation, Armonk, NY, USA) and GraphPad Prism (version 8.3.0 for Windows, GraphPad Software, La Jolla, CA, USA).

## Results

### Demographics and resection of RCC

The 40 patients analyzed underwent nephrectomy due to histologically confirmed RCC in curative intent. The median age at the time of nephrectomy was 54 years (35–80). Left and right nephrectomies were equally distributed among the patient cohort; one patient received bilateral nephrectomy.

Further demographic, clinical, and histopathological data are displayed in Supplemental Table 1 (Online Resource 1).

### Hepatic metastasectomy

The median interval between nephrectomy for RCC and hepatic metastasectomy was 44 months (3.3–278.5). Of note, three patients displayed synchronous (but isolated) hepatic metastases and received partial hepatectomy immediately after nephrectomy.

**Fig. 1:**
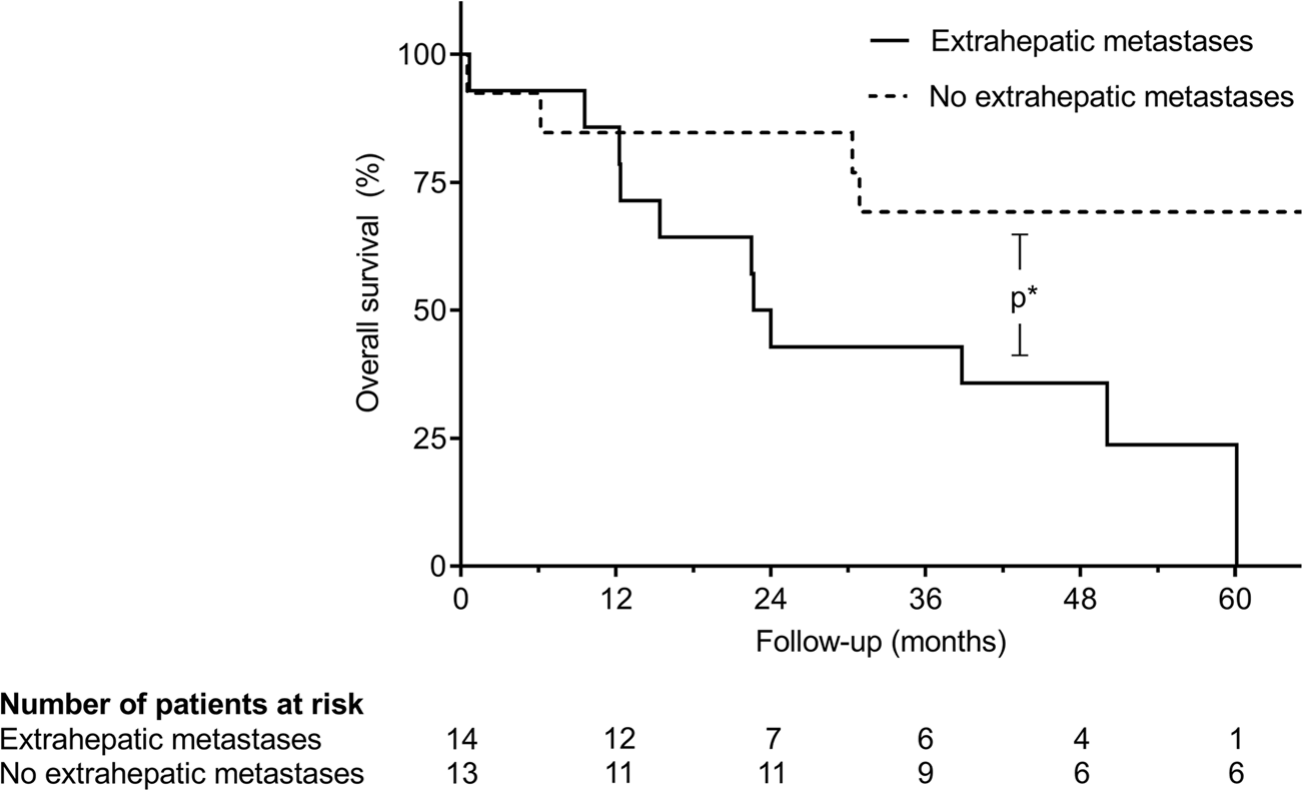
Kaplan-Meier survival analysis after hepatic metastasectomy in cases of concomitant extrahepatic distant metastases: OS after resection of hepatic metastases in cases of concomitant distant metastases was significantly worse than in patients with isolated liver metastases (p* = 0.017)

Fourteen patients were perioperatively diagnosed with multiple metastatic sites including ossary, pulmonary, and further intraabdominal lesions and showed inferior median DFS and OS (9.9 and 23.4 months) when compared with patients without extrahepatic metastases (23.5 and 47.2 months; *p* = 0.062 and 0.017, respectively; Fig. 1).

Localization of liver metastases was mainly observed within the right liver lobe (57.5%). However, 15 patients (37.5%) suffered from bilateral metastases, resulting in a shorter median DFS (8.4 versus 19.7 months; *p* = 0.562) and OS (24.0 months versus 41.5 months; *p* = 0.809) by trend.

Accordingly, right or right extended hemihepatectomy was most frequent among the resections performed (12 patients (30.0%) and 8 patients (20.0%)).

**Fig. 2:**
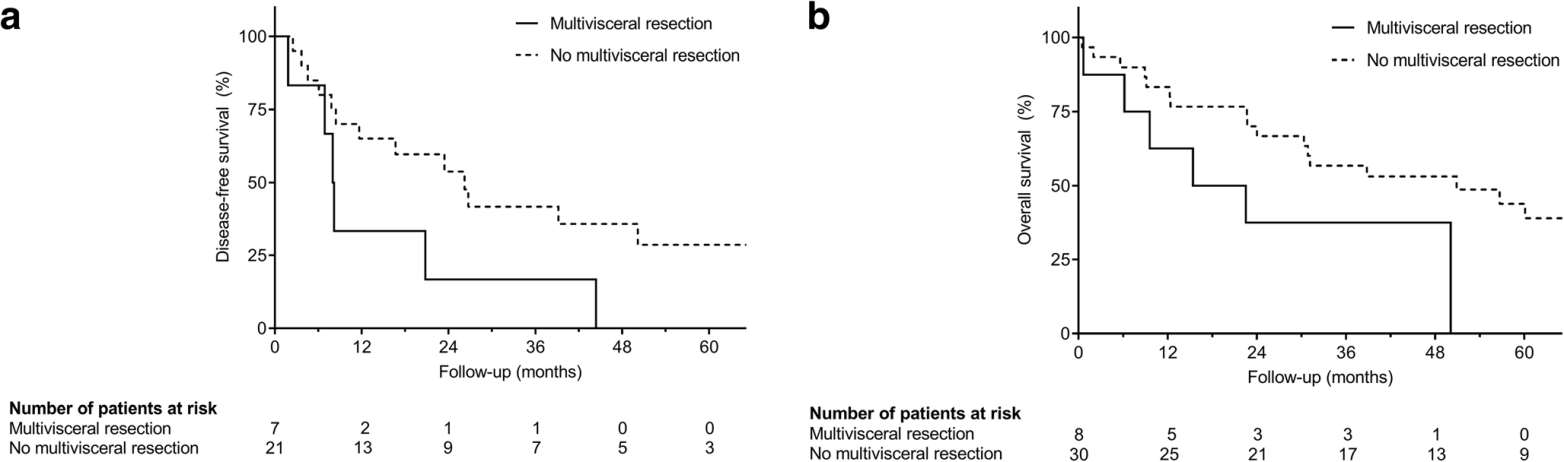
Kaplan-Meier survival analysis after hepatic metastasectomy in cases of multivisceral resection: patients undergoing simultaneous multivisceral resection showed inferior DFS (**a***p* = 0.060) and OS (**b***p* = 0.096) when compared with patients undergoing isolated liver resection

Multivisceral resections were performed in 8 patients (20.0%). These included additional partial pancreatic resections (4 patients) alongside an adrenalectomy (1 patient) and partial resections of the retrohepatic vena cava (2 patients), the diaphragm (2 patients), and the transverse colon (1 patient) due to tumor invasion. Only one of these patients died in the postoperative course; however, median DFS (8.0 versus 19.7 months; *p* = 0.060; Fig. 2a) and OS (19.0 versus 41.0 months; *p* = 0.096; Fig. 2b) were lower when compared with all other patients.

Median follow-up after hepatic metastasectomy was 37.8 months (0.5–286.5).

The distribution of demographic and clinical variables of the patients undergoing hepatic metastasectomy is presented in Table 1a.

**Table 1:** (a) Clinical and histopathological variables of the 40 patients undergoing hepatic metastasectomy. (b) Multivariable cox regression analysis for OS

(a)				
Variables		*n* (%)	Mean, median (min.–max.)	HR; 95%CI; *p* value
Age at metastasectomy (in years)		n.a.	60.9, 60 (38–80)	0.997; 0.960–1.035; 0.869
Male gender		25 (62.5)	n.a.	1.483; 0.660–3.330; 0.340
Synchronous hepatic metastases		3 (7.5)	n.a.	0.034; 0.000–4.989; 0.184
Interval between nephrectomy and metastasectomy (in months)		n.a.	62.3, 44 (3.3–278.5)	0.988; 0.977–1.000; 0.044
Interval between nephrectomy and metastasectomy (less than 12 months)		8 (20.0)	n.a.	1.135; 0.420–3.069; 0.803
Extrahepatic metastases		14 (50.0)	n.a.	3.847; 1.184–12.494; 0.025
Modified Memorial Sloan-Kettering Prognostic Score	Favorable-risk group	19 (61.3)	n.a.	0.853; 0.346–2.101; 0.729
	Intermediate-risk group	8 (25.8)		0.873; 0.315–2.425; 0.795
	Poor-risk group	4 (12.9)		1.918; 0.534–6.888; 0.318
Localization of hepatic metastases	Right	23 (57.5)	n.a.	1.059; 0.479–2.342; 0.888
	Left	2 (5.0)		1.334; 0.178–10.015; 0.779
	Bilateral	15 (37.5)		0.905; 0.402–2.037; 0.809
Type of liver resection	Wedge resection	6 (15.0)	n.a. n.a.	
	Segmental resection	12 (30.0)		
	Right hemihepatectomy	12 (30.0)		
	Left extended	1 (2.5)		
	Right extended	8 (20.0)		
	Ante situ resection	1 (2.5)		
Extent of surgery	Major hepatectomy	24 (60.0)	n.a.	0.760; 0.346–1.668; 0.494
	Extended hepatectomy	9 (22.5)	n.a.	1.027; 0.439–2.401; 0.952
	Multivisceral resection	8 (20.0)	n.a.	2.223; 0.848–5.830; 0.104
Operation time (in minutes)		n.a.	231.5, 210 (45–670)	1.005; 1.001–1.010; 0.010
Portal occlusion (in minutes)		n.a.	33.1, 25 (0–220)	1.011; 0.998–1.024; 0.101
Pathology	Multiple hepatic metastases	18 (48.6)	n.a.	0.413; 0.178–0.959; 0.040
	Number of metastases	n.a.	2.3, 1 (1–12)	0.726; 0.516–1.022; 0.066
	Diameter of metastases (in mm)	n.a.	48.8, 42.5 (1.5–150)	1.001; 0.988–1.015; 0.885
	Positive resection margins (R1)	3 (7.5)	n.a.	2.452; 0.562–10.690; 0.223
	Safety margins (in mm)	n.a.	8.6, 5 (1–35)	1.016; 0.971–1.064; 0.487
Before July 2006 (approval of sunitinib in Europe)		25 (62.5)	n.a.	2.718;1.017–7.265; 0.046
(b)				
Variables		HR	95%CI	*p* value
Interval between nephrectomy and metastasectomy (in months)		0.971	0.956–0.987	< 0.001
Multivisceral resection		9.851	2.715–35.737	0.001

Clinical and histopathological variables of the 40 patients undergoing hepatic metastasectomy including the results from univariable Cox regression analysis for OS (*HR* hazard ratio, *CI* confidence interval)

Multivariable analysis identified a longer interval between nephrectomy as an independent protective factor and multivisceral resection as an independent risk factor for OS

### Histopathological results

Postoperative histopathology of the liver specimens revealed tumor-free resection margins in 37 patients (92.5%). Although a trend toward better OS was observed, further analysis did not reveal a significant influence of tumor-free resection margins on survival (38.8 versus 6.2 months; *p* = 0.217).

The histopathological results of the patients undergoing hepatic metastasectomy are summarized in Table 1a.

### Postoperative outcome

Postoperative complications of various extent occurred in 16 patients (41.0%), with 9 patients (23.1%) suffering from severe complications (≥ Clavien-Dindo grade III) including two patients dying in the ICU due to post-hepatectomy liver failure and postoperative hemorrhage, respectively.

The incidence of postoperative complications in general and severe complications was not significantly elevated with respect to the extent of hepatic surgery; however, multivisceral resections led to a significant increase of postoperative complications in general (75.0% versus 32.3%, *p* = 0.028) and severe complications (62.5% versus 12.9%, *p* = 0.003).

**Fig. 3:**
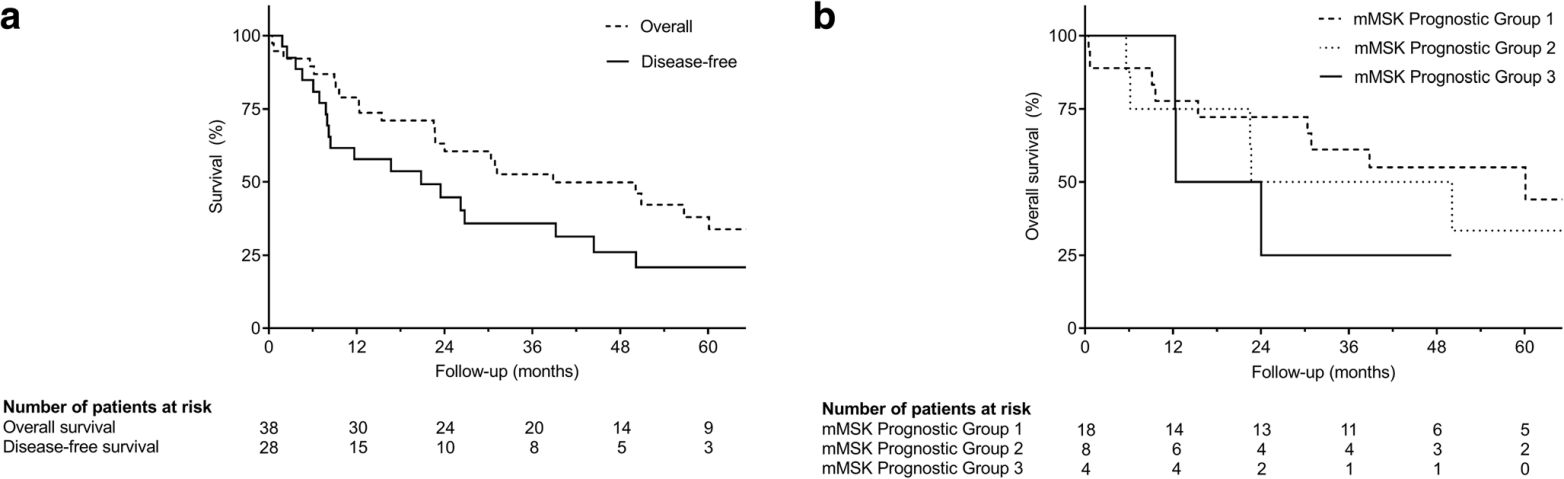
Kaplan-Meier analysis of DFS and OS after resection of hepatic metastases (**a**) and of OS according to the modified Memorial Sloan-Kettering (mMSK) prognostic groups (**b**): survival of patients allocated to the poor prognostic group (3) was worse than in patients allocated to the favorable (1) and intermediate prognostic groups (2); however, without statistical significance (*p* = 0.595)

Tumor recurrence was observed in 19 patients (67.9%) resulting in a median DFS of 16.2 months (0.7–265.1; Fig. 3a).

Median OS was 37.8 months (0.5–286.5), whereas 28 patients (73.7%) were deceased at the time of analysis (Fig. 3a).

The prognostic group classification as defined by the modified Memorial Sloan-Kettering Score was associated with inferior survival, but not in a statistically significant manner (*p* = 0.595; Fig. 3b).

Table 2 summarizes the postoperative outcome after hepatic metastasectomy.

**Table 2:** Postoperative outcome after hepatic metastasectomy

Variables		*n* (%)	Mean, median (min.–max.)
ICU stay (in days)		n.a.	5.4, 1 (1–49)
Hospital stay (in days)		n.a.	22.7, 15 (10–92)
Postoperative complications		16 (41.0)	n.a.
Postoperative complications (classified by Clavien-Dindo)	0	23 (59.0)	n.a.
	I	3 (7.7)	
	II	4 (10.3)	
	III	6 (15.4)	
	IV	1 (2.6)	
	V	2 (5.1)	
Follow-up (in months)		n.a.	50.8, 37.8 (0.5–286.5)
Tumor recurrence		19 (67.9)	n.a.
DFS (in months)		n.a.	36.4, 16.2 (0.7–265.1)
	Minor hepatic resection	n.a.	17.5, 11.7 (3.7–50.2)
	Major hepatic resection	n.a.	48.7, 23.5 (0.7–265.1)
	Extended hepatic resection	n.a.	49.1, 8.4 (0.7–154.3)
	Multivisceral resection	n.a.	13.0, 8.0 (0.7–44.4)
OS (in months)		n.a.	50.8, 37.8 (0.5–286.5)
	Minor hepatic resection	n.a.	37.8, 43.2 (6.2–111.0)
	Major hepatic resection	n.a.	59.3, 36.8 (0.5–286.5)
	Extended hepatic resection	n.a.	62.0, 31.2 (0.7–205.8)
	Multivisceral resection	n.a.	24.2, 19.0 (0.7–50.1)
1-year OS (Kaplan-Meier)		78.9	n.a.
3-year OS (Kaplan-Meier)		52.6	n.a.
5-year OS (Kaplan-Meier)		38.0	n.a.
Dead at time of analysis		28 (73.7)	n.a.

### Approval of sunitinib in Europe

After introduction of the tyrosine kinase inhibitor sunitinib in Europe in July 2006, 15 patients underwent hepatic metastasectomy. Of these, six patients received targeted therapy either pre- or postoperatively, with sunitinib being initially administered to all patients. Due to severe side effects in two patients, targeted therapy was switched consecutively to other agents, such as pazopanib, temsirolimus, everolimus, and lenvatinib. Of note, none of the patients included in this study received targeted therapy prior to official approval of sunitinib in Europe, neither off-label nor in the course of clinical studies.

**Fig. 4:**
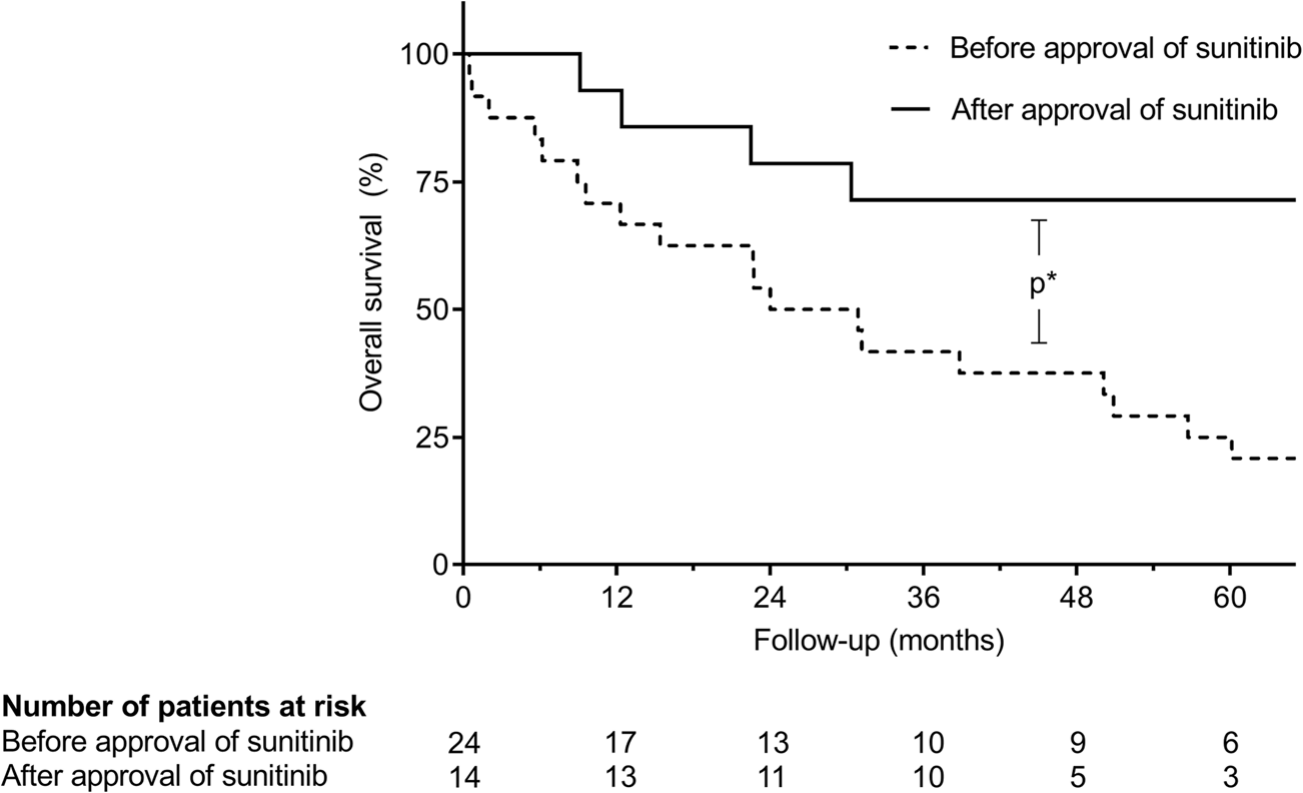
Kaplan-Meier analysis of OS after resection of hepatic metastases before and after approval of sunitinib: survival after hepatic metastasectomy before approval of sunitinib in July 2006 was significantly worse than in patients after July 2006 (p* = 0.038)

Median OS after July 2006 increased significantly (45.2 versus 27.5 months, *p* = 0.038; Fig. 4), although patients were significantly older at the time of liver resection (mean age 66.9 versus 57.2, *p* = 0.002).

The extent of hepatic resections by trend was smaller after 2006, with rates of major and extended hepatic resections at 46.7% (versus 68.0%, *p* = 0.182) and 6.7% (versus 32.0%, *p* = 0.063), respectively, also reflected by a significantly shorter mean operation time (165.3 versus 263.3, *p* = 0.014). Of note, the extent of surgery did not significantly affect DFS or OS in patients undergoing hepatic metastasectomy after introduction of sunitinib.

None of the patients undergoing liver resection after 2006 died in the peri- or postoperative course.

A statistical comparison of selected variables before and after approval of sunitinib is given in Table 3.

**Table 3:** Comparison of selected variables at the time of hepatic metastasectomy and postoperative outcome before and after approval of sunitinib in 2006

Variables			Before July 2006 Mean, median (min.–max.) or *n* (%)	After July 2006 Mean, median (min.–max.) or *n* (%)	*p* value
Age at metastasectomy (in years)			57.2, 59 (38–75)	66.9, 68 (57–80)	0.002
Male gender			17 (68.0)	8 (53.3)	0.354
Synchronous hepatic metastases			1 (4.0)	2 (13.3)	0.278
Interval between nephrectomy and metastasectomy (in months)			50.6, 25.9 (3.3–216.9)	85.7, 48.0 (6.3–278.5)	0.159
Modified Memorial Sloan-Kettering Prognostic Score	Favorable-risk group		9 (56.3)	10 (66.7)	0.771
	Intermediate-risk group		5 (31.3)	3 (20.0)	
	Poor-risk group		2 (12.5)	2 (13.3)	
Localization of hepatic metastases	Right		12 (48.0)	11 (73.3)	0.208
	Left		1 (4.0)	1 (6.7)	
	Bilateral		12 (48.0)	3 (20.0)	
Type of liver resection	Wedge resection		1 (4.0)	5 (33.3)	0.148
	Segmental resection		8 (32.0)	4 (26.7)	
	Right hemihepatectomy		7 (28.0)	5 (33.3)	
	Left extended		1 (4.0)	0 (0.0)	
	Right extended		7 (28.0)	1 (6.7)	
	Ante situ resection		1 (4.0)	0 (0.0)	
Extent of surgery	Major hepatectomy		17 (68.0)	7 (46.7)	0.182
	Extended hepatectomy		8 (32.0)	1 (6.7)	0.063
	Multivisceral resection		5 (20.0)	3 (20.0)	1.000
Operation time (in minutes)			263.3, 250.0 (45–670)	165.3, 145.5 (104–344)	0.014
Portal occlusion (in minutes)			41.6, 29.5 (0–220)	16.1, 10.0 (0–89)	0.125
Pathology	Multiple hepatic metastases		10 (40.0)	8 (53.3)	0.420
	Number of metastases		1.8, 1 (1–5)	3.3, 2 (1–12)	0.388
	Diameter of metastases (in mm)		53.1, 42.5 (1.5–150)	41.5, 33.5 (7–120)	0.327
	Positive resection margins (R1)		2 (8.0)	1 (6.7)	0.877
	Safety margins (in mm)		11.2, 8 (1–35)	4.1, 3 (1–12)	0.085
Postoperative outcome	ICU stay (in days)		6.2, 1 (1–49)	3.9, 1 (1–32)	0.467
	Hospital stay (in days)		24.1, 15 (10–92)	21.6, 15 (10–60)	0.970
	Postoperative complications		10 (40.0)	6 (42.9)	0.862
	Postoperative complications (classified by Clavien-Dindo)	0	15 (60.0)	8 (57.1)	0.601
		I	1 (4.0)	2 (14.3)	
		II	3 (12.0)	1 (7.1)	
		III	3 (12.0)	3 (21.4)	
		IV	1 (4.0)	0 (0.0)	
		V	2 (8.0)	0 (0.0)	
	Follow-up (in months)		53.2, 27.5 (0.5–286.5)	46.7, 45.2 (9.1–111)	0.402
	Tumor recurrence		9 (64.3)	10 (71.4)	0.686
	DFS (in months)		54.9, 20.1 (0.7–265.1)	18.0, 13.7 (1.8–58.3)	0.303
	OS (in months)		53.2, 27.5 (0.5–286.5)	46.7, 45.2 (9.1–111)	0.038
	Dead at time of analysis		23 (95.8)	5 (35.7)	< 0.001

### Identification of risk and protective factors for survival

Univariable Cox regression analysis did not reveal significant risk factors for DFS. However, patients with extrahepatic disease and multivisceral resection showed a trend toward inferior DFS (Table 4a). Multivariable analysis identified multivisceral resection as an independent risk factor (HR, 2.966; 95%CI, 1.035–8.495; *p* = 0.043) for DFS (Table 4b).

**Table 4:** (a) Univariable Cox regression analysis for DFS. (b) Multivariable Cox regression analysis for DFS

(a)				
Variables		HR	95%CI	*p* value
Age at metastasectomy (in years)		0.993	0.948–1.040	0.768
Male gender		1.846	0.698–4.877	0.216
Interval between nephrectomy and metastasectomy (in months)		0.997	0.990–1.005	0.512
Extrahepatic metastases		2.520	0.924–6.872	0.071
Bilateral localization of hepatic metastases		0.750	0.282–1.992	0.564
Extent of surgery	Major hepatectomy	0.543	0.216–1.363	0.194
	Extended resection	0.547	0.158–1.889	0.340
	Multivisceral resection	2.512	0.931–6.782	0.069
Operation time (in minutes)		1.001	0.995–1.007	0.782
Portal occlusion (in minutes)		0.971	0.941–1.002	0.067
Pathology	Multiple hepatic metastases	0.947	0.326–2.748	0.920
	Number of metastases	0.905	0.745–1.099	0.312
	Diameter of metastases (in mm)	0.996	0.979–1.014	0.664
	Positive resection margins	1.009	0.132–7.706	0.993
	Safety margins (in mm)	1.016	0.955–1.081	0.608
Before July 2006 (introduction of sunitinib in Europe)		0.618	0.245–1.557	0.307
(b)				
Variables		HR	95%CI	*p* value
Multivisceral resection		2.966	1.035–8.495	0.043

Univariable Cox regression analysis did not reveal significant risk factors for DFS; however, patients with extrahepatic disease and multivisceral resection showed a trend toward inferior DFS

Multivisceral resection was identified as independent significant risk factor for DFS

The presence of extrahepatic metastases, longer operating times, and hepatic metastasectomy before July 2006 were identified as risk factors for OS, whereas a longer interval between nephrectomy and hepatic metastasectomy and the presence of multiple hepatic lesions were identified as protective factors for OS in univariable analysis (Table 1a).

Multivariable analysis identified a longer interval between nephrectomy as an independent protective factor (HR, 0.971; 95%CI, 0.956–0.987; *p* < 0.001) and multivisceral resection as an independent risk factor (HR, 9.851; 95%CI, 2.715–35.737; *p* = 0.001) for OS (Table 1b).

## Discussion

Despite several studies on metastasectomy in patients suffering from grade IV RCC, the role of resection of hepatic lesions, especially in times of efficient systemic therapy, remains elusive.

In the current study, we present the largest monocentric database on the matter since Staehler et al. published their patient series in 2010 and, to our knowledge, the first series investigating patient survival following hepatic metastasectomy in the era of targeted therapy [14].

We found a median OS of 37.8 months with 1-, 3-, and 5-year survival rates (78.9%, 52.6%, and 38.0%, respectively) in line with results reported in a review by Pikoulis and more recently Pinotti et al. [7, 8].

The published heterogeneous survival (16–142 months) is partially explained by the varying rates of simultaneous extrahepatic disease (0–37%) and the varying rates of major liver resections in reported patient series (14–85.7%) [14–17]. In our study, extrahepatic metastases were observed in 14 patients (35.0%) and led to a significantly worse OS, as was reported by others in the past [17, 18]. The proportion of patients undergoing major hepatic surgery in our series was also comparatively large (60.0%). However, major or extended liver resections (22.5%) were not associated with inferior patient outcome, and although multivisceral resection (20.0%) was identified as an independent significant risk factor for inferior DFS and OS, the median OS in these patients was still considerable (19 months). Of note, multivisceral resections included pancreatic metastases since beneficiary survival has been shown in cases of pancreatic metastasectomy by others in the past [19, 20].

Even though high-volume oncologic liver surgery is performing at our institution, our observation period was long, spanning three decades due to the low incidence of resectable RCC liver metastases, like in most reports on the matter [7, 8, 21]. Since surgical protocols were refined and, even more importantly, systemic therapy has improved over time, we have analyzed our patient series according to the introduction of the tyrosine kinase inhibitor sunitinib for stage IV RCC in Europe in July 2006, taking into account the significant efficiency of targeted therapy [22–24].

Despite entering the era of targeted therapy, surgical resection is still regarded as the only chance for long-time survival of metastatic RCC [4, 5]. Accordingly, results from a study of McKay et al. investigating the outcome of metastasized RCC with liver metastases (alone or in combination with extrahepatic sites) under targeted therapy showed a median OS of up to 18.2 months, which is significantly inferior to the outcome reported in the current study. However, the authors did not comment on patient factors excluding surgical resection as an additional or alternative therapeutic strategy [25].

Although several reports of beneficial effects of metastasectomy in combination with targeted therapy were published in the past, the specific role of hepatic metastasectomy remains unclear, since only few studies examining the latter included patients resected in the era of targeted therapy [5, 8, 26]. To our knowledge, Staehler et al. were the first and to date only group of authors, that included a control group receiving systemic therapy instead of surgical resection. However, the study was performed before wider use of targeted therapy such as sunitinib, with only 5 patients receiving targeted therapy [14].

In our patient series, the outcome in general significantly improved in case of hepatic metastasectomy after July 2006. Nonetheless, multivariable analysis did not show an independent significant influence of hepatic metastasectomy after July 2006 on OS. This could be partially due to the fact, that the maximum follow-up time was shorter in this subgroup, with 10 patients (64.3%) still being alive at the time of analysis as opposed to only one patient (4.2%) out of the subgroup undergoing liver surgery before introduction of targeted therapy. Another potential confounder observed was a longer disease-free interval between primary nephrectomy and liver resection, known as an independent significant factor for better survival, in patients undergoing hepatic metastasectomy after July 2006 (48.0 versus 25.9 months) [15–18, 27, 28]. Furthermore, a tendency toward lesser extended hepatic surgery, reflected by significantly shorter operating times and lower rates of major and extended liver resections (46.7% versus 68.0%), since the introduction of targeted therapy was found. Despite these observations, we cannot prove that solely the option of targeted therapy led to less (aggressive) hepatic surgery in our patients, due to the retrospective nature of the study. Other developments in the treatment of hepatic malignancies, such as the introduction and widespread use of less invasive technologies, including radiofrequency ablation, selective internal radiation therapy, or transarterial chemoembolization, also have to be discussed as an explanation for our findings.

We analyzed further factors supposedly influencing postoperative outcome. These included positive resection margins which were associated with a higher hazard ratio but did not significantly influence patient survival, as opposed to several reports in the past [27–30]. An explanation could be the small number of only three patients showing positive resection margins, reflecting an aggressive approach and resulting in a comparatively high rate of major and extended hepatic and multivisceral resections.

As summarized by the review from Pikoulis et al., neither the number, the size, nor the laterality of liver metastases had an impact on patient outcome [7].

The modified Memorial Sloan-Kettering Prognostic Factors Model for patients with metastasized RCC could not be validated in our patient series, despite a trend toward superior survival for the favorable-risk group. This could be the result of a comparatively small sample size, with only four patients allocated to the poor-risk group. We suggest that larger patient series, preferably in multi-center studies, should be analyzed in the future to clarify the role of the abovementioned prognostic model in patients with RCC liver metastases.

Limitations of the present study are the retrospective nature combined with a heterogeneous patient selection and missing control groups, diminishing the statistical power of our observations. Of note, collection of complete clinical and histopathological data of patients undergoing hepatic metastasectomy, especially in the early observation period, was partially not possible since patients, relatives, and even physicians in charge were deceased at the time of analysis. We believe, however, that it is essential to report on patient series with a comparatively rare surgical indication and that our findings could contribute to facilitating optimal patient selection and subsequent outcome in the future of a rapidly evolving therapeutic landscape [31].

## Conclusion

Despite the introduction of novel targeted therapies, surgical concepts still remain the only chance for long-term survival or cure in selected patients. Hepatic metastasectomy on patients with RCC stage IV disease can be performed safely and allows long-term survival, albeit with an increased risk of postoperative complications especially following multivisceral surgery. It will be crucial to define the role of hepatic metastasectomy with a focus on patients suffering from an extended metastatic disease in the context of future multimodal therapy strategies.

## Electronic supplementary material

ESM 1 (PDF 277 kb)
