# Supplementary material for: Extended hepatic metastasectomy for renal cell carcinoma—new aspects in times of targeted therapy: a single-center experience over three decades
Source: Langenbecks Arch Surg. 2020 Jan 14;405(1):97–106. doi: 10.1007/s00423-019-01852-4 (PMC7036059; doi:10.1007/s00423-019-01852-4)
Supplement: Supplementary file 1 — (PDF 277 kb) [file 423_2019_1852_MOESM1_ESM.pdf]

Extended hepatic metastasectomy for renal cell carcinoma – new aspects in times of targeted therapy: a single center experience over three decades

Langenbeck's Archives of Surgery

Oliver Beetz, Rabea Söffker, Sebastian Cammann, Felix Oldhafer, Florian W. R. Vondran, Florian Imkamp, Jürgen Klempnauer, Moritz Kleine

Correspondence and reprint requests to:

PD Dr. med. Moritz Kleine, Department of General, Visceral and Transplant Surgery, Hannover Medical School, Carl-Neuberg-Strasse 1, 30625 Hannover, Germany

(e-mail: Kleine.Moritz@mh-hannover.de; phone: +49-511-532-6534; fax: +49-511-532-4010)

ORCID: 0000-0002-4515-3209

Supplemental Table 1 Epidemiological, clinical and histopathological statistics of the 40 patients undergoing nephrectomy for renal cell carcinoma in curative intent

Variables		Mean, Median (min.-max.) or n (%)
	**Age at nephrectomy (in years)**	55.8, 54 (35-80)
	**Male gender**	25 (62.5)
**Localization RCC**	right	16 (48.5)
	left	16 (48.5)
	bilateral	1 (3.0)
**T stadium**	1	3 (12.5)
	2	6 (25.0)
	3	13 (54.2)
	4	2 (8.3)
**N stadium**	0	5 (26.3)
	1	10 (52.6)
	2	4 (21.1)
**M stadium**	0	9 (42.9)
	1	8 (38.1)
	2	4 (19.0)
	**Clear cell renal carcinoma**	16 (94.1)
	**Chemotherapy**	13 (59.1)
	**Radiotherapy**	5 (29.4)
